# Transcriptome-microRNA analysis of *Sarcoptes scabiei* and host immune response

**DOI:** 10.1371/journal.pone.0177733

**Published:** 2017-05-23

**Authors:** Ran He, Xiaobin Gu, Weimin Lai, Xuerong Peng, Guangyou Yang

**Affiliations:** 1 Department of Parasitology, College of Veterinary Medicine, Sichuan Agricultural University, Wenjiang, China; 2 Department of Chemistry, College of Life and Basic Science, Sichuan Agricultural University, Wenjiang, China; Agency for Science Technology and Research, SINGAPORE

## Abstract

Scabies is a parasitic disease, caused by the mite *Sarcoptes scabiei*, and is considered one of the top 50 epidemic diseases and one the most common human skin disease, worldwide. Allergic dermatitis, including an intense itch, is a common symptom, however diagnosis is difficult and there is currently no effective vaccine. The goal of this study was to examine the immune interaction mechanism of both *S*. *scabiei* and infected hosts. mRNA-seq and microRNA-seq were conducted on the *S*. *scabiei* mite and on infected and uninfected hosts. We focused on differential expression of unigenes and microRNAs, as well as the real targets of unigenes in enriched immune signaling pathways. *S*. *scabiei* enhanced host immune function and decreased metabolism after infection, while the immune response of the host inhibited *S*. *scabiei* proliferation and metabolism signaling pathways. Differentially expressed unigenes of *S*. *scabiei* were enriched in the JAK-STAT signaling pathway and the Toll-like receptor signaling pathway. The differential expression analysis indicated that microRNAs of *S*. *scabiei* and hosts have major roles in regulating immune interactions between parasites and hosts.

## Introduction

Scabies, caused by the parasitic mite *Sarcoptes scabiei*, is an allergic skin disease that affects millions of people and mammals worldwide[1]. *S*. *scabiei* has a broad host range, capable of infecting more than 100 species livestock and wild animals. It is listed as a neglected tropical disease by the World Health Organization[1, 2], and is considered one of the top 50 epidemic diseases worldwide. Additionally, it is one of the most common skin diseases in children[3–5]. Diagnosis is based on clinical characteristics; however, asymptomatic patients are often misdiagnosed[6]. Scabies presents a significant public health burden, particularly in economically disadvantaged populations. Additionally, scabies could cause secondary bacterial infections, which often followed by a series of other diseases, streptococcal infection for example, resulting in chronic kidney disease[7–9]. *S*. *scabiei* mite could burrow into skins and results in itching, epilation, and crusting of the wound for host[10, 11]. *S*. *scabiei* obtains nutrients from epidermal cells and serum of its host[1], and infection may lead to loss of appetite, weight loss and death of the host. *S*. *scabiei* also causes a global economic burden in animals[12].

Previous research has focused primarily on diagnostic methods, acaricidal drugs, recombinant protein antigen vaccines, and allergen proteins, such as cytokines and antibodies[8, 12–30]. There is currently no effective vaccine for the scabies mite, and treatments for animals include application of chemical acaricides have resulted in resistance in target species and environmental pollution[31]. Research exploring the immunopathogenesis and allergens of the scabies mite, and host immunological protective mechanisms is warranted as these genes are potential targets for vaccine development or are potential markers for diagnostic tests[12, 31, 32].

In this study, we examined the transcriptome and microRNAs in response to infection with *S*. *scabiei* in order to resolve our understanding of the immune interaction between this mite and a host species. This study explored the immune response of *S*. *scabiei* and host with transcriptome and microRNA comprehensive analysis and provided a resource for the further research.

## Methods

### Host infection with *Sarcoptes scabiei*

New Zealand rabbits were provided by the Laboratory Animal Center of Sichuan Agricultural University (Ya’an, China). Nine New Zealand rabbits were separated into three groups randomly before infection, and all rabbits were housed under a barrier environment in sterile cages and provided with pelleted food and sterilized water ad libitum. Rabbits were acclimated to these conditions for one week prior to the experiment. Scabies mites (including adult, nymphs and larvae) were provided by Sichuan Agriculture University’s Parasitology Laboratory. Two groups of rabbits were infected with same weight mites (approximately 2000 live mites) on the surface of four feet and wrapped by gauze for 48h. For the first group, the mites were collected and held starved for 12 h before being stored in liquid nitrogen. For the second group, the mites, and the skin (with scraping) surrounding them, were collected and immediately stored in liquid nitrogen. The last group, uninfected rabbits were used as a negative control. Skin from the rabbits feet were collected and stored in liquid nitrogen (S1 Table).

All rabbits used in this study were 120 days old and weighed between 3–3·25 kg.

The Animal Ethics Committee of Sichuan Agricultural University (AECSCAU; Approval No. 2011–028) had reviewed and approved this study. Animals were handled strictly according to the animal protection law of the People’s Republic of China (released on 09/18/2009) and the National Standards for Laboratory Animals in China (executed on 05/1/2002).

### Isolation of total RNA

Total RNAs were extracted using a single step acid guanidinium thiocyanate-phenol-chloroform method with modifications. Samples (starved mites, embedded mites with infected host skin, uninfected host skin) were pulverized by fine grinding in liquid nitrogen and were transferred to an ice-chilled guanidinium thiocyanate mix. After a 5 min incubation on ice, followed by centrifugation at 4°C, the RNA-containing supernatants were transferred to a new tube and ethanol precipitation was conducted, followed by washing and dissolving in DEPC-treated water. The RNAs were further cleaned by two phenol-chloroform treatments. RNA was treated with RQ1 DNase (Promega) to remove DNA. Purity and integrity were assessed by agarose gel electrophoresis (1.5%) and quantified by measuring the absorbance at 260/280 nm on a SmartSpec (Bio-rad).

### Library construction and transcriptome sequencing

For each sample, 10 μg of total RNA was used for RNA-seq library preparation. Polyadenylated mRNAs were purified and concentrated with oligo (dT)-conjugated magnetic beads (Invitrogen) before used in directional RNA-seq library preparation. Purified mRNAs were iron fragmented at 95°C followed by end repair and 5' adaptor ligation. Then reverse transcription was performed with RT primers harboring 3' adaptor sequence and a randomized hexamer. The cDNAs were purified and amplified and PCR products corresponding to 200–500 bps were purified, quantified and stored at -80°C until used for sequencing. The libraries were prepared with a RNA-Seq Library Preparation Kit for Whole Transcriptome Discovery (GnomeGen, San Diego, CA, USA) following the manufacturer's instructions and sequenced on an Illumina Hiseq 2000 system (Illumina, San Diego, CA, USA) using 2x100-bp paired-end sequencing.

The total RNA (3 μg) was used for microRNA cDNA library preparation with a Balancer NGS Library Preparation Kit for small/microRNA (GnomeGen, San Diego, CA, USA) following manufacturer's instruction. RNAs were ligated to 3' and 5' adaptor sequentially, reverse transcribed to cDNA and PCR amplified. Whole library was applied to 10% native PAGE gel electrophoresis and bands corresponding to microRNA insertion were cut and eluted. After ethanol precipitation and washing, the purified small RNA libraries were quantified with Qubit Fluorometer (Invitrogen) and used for cluster generation and 36 nt single end sequencing analysis through the Illumina GAIIx (Illumina, San Diego, CA, USA).

### RNA-seq data processing

Raw reads were discarded if they contained more than 2-N bases, then were processed by removing the adaptor sequence and low quality bases, and discarding reads less than 16nt using FASTX-Toolkit v 0.0.13 FastQC (http://www.bioinformatics.babraham.ac.uk/projects/fastqc)[33]. Sequences obtained from rabbit samples were aligned to the European rabbit’s (*Oryctolagus cuniculus*) genome from Genbank (http://www.ncbi.nlm.nih.gov/Taxonomy/taxonomyhome.html/index.cgi?chapter=howcite) using TopHat2[34]. Reads obtained from starved and skin-embedded *S*. *scabiei* were combined for analysis. *S*. *scabiei* sequences were assembled using Trinity[35] and annotation of the assembled transcript sequences was performed using BLASTX algorithm and non-redundant protein database on NCBI, Nt and the COG databases (http://www.ncbi.nlm.nih.gov/). An e-value of 1e-5 was designated as the cutoff[36]. *S*. *scabiei* clean reads were aligned to unigenes and aligned reads with more than one genome location were considered ambiguous and discarded. Uniquely localized reads were used to calculate read number and RPKM value (reads per kilobase and per million) for each gene based on reads and location of genes within the genome. Gene coverage and depth, and read distribution around start codon and stop codon, were determined.

### Functional enrichment analysis

Differentially expressed genes and unigenes were submitted to the Database for Annotation, Visualization and Integrated Discovery (DAVID:https://david.ncifcrf.gov/) for enrichment analysis[37]. Enrichment clusters were sorted by the enrichment score in descending order and categories within clusters were sorted by p-value in descending order. Fold enrichment with Bonferroni, Benjamini, and FDR corrected p-values were calculated for each category of each cluster.

### MicroRNA-seq data processing

Raw reads were discarded if they contained more than 2-N bases, and were processed by removing the adaptor, low quality bases, and reads less than 12nt using FASTX-Toolkit v 0.0.13. FastQC (http://www.bioinformatics.babraham.ac.uk/projects/fastqc) was used to verify post-filtering read quality[38].

MicroRNA-seq clean reads were aligned to the RNA family database (Rfam, v 11.0)[39] and the microRNA database (miRBase, v19)[40] using Bowtie[41] with no more than 2-nt mismatch. Only sequences in this study were selected. Both mature and hairpin microRNA reference sequences were used. First reads were aligned to mature microRNA sequences, and then unmapped reads were aligned to reference hairpin sequences. Read number and tag per million (TPM) values were obtained from the alignment. For each microRNA, we predicted the target unigene using mature microRNA sequences and whole mRNA sequences (include coding sequences and untranslated regions) with the program Miranda[42].

### Differentially expressed genes

Differentially expressed genes (DEG) and unigenes from both RNA and microRNA sequences were analyzed to compare infected rabbit test samples and uninfected rabbit control samples, using the R package, edgeR[43] based on the negative binomial distribution. Thresholds of p < 0.01 and a 2-fold change were set to define DEGs.

### RNA-seq and microRNA-seq comprehensive analysis

Using the predicted target unigenes from microRNA sequences and the differentially expressed unigenes, we filtered the target unigenes using Miranda[42]. The potential target unigenes and mRNA-seq DEG overlap were analyzed for possible real target unigene. In order to understand the function of DE-microRNA target unigenes, we used the DAVID (https://david.ncifcrf.gov/) online platform[37].

### Allergen and hydrolase genes

To examine homologous allergen gene sequences between *S*. *scabiei* and three other mite species, 287 allergen genes from *Dermatophagoides farina*, *Dermatophagoides pteronyssinus*, and *Euroglyphus mayne*, were downloaded from NCBI (http://www.ncbi.nlm.nih.gov/). Additionally, we compared our mRNA-seq results with hydrolase genes by building a *S*. *scabiei* hydrolase gene library with the best match of homologous sequences.

### Validation of mRNA-seq and microRNA-seq results by RT-PCR

According to the comprehensive analysis of mRNA and microRNA results, we validated the unigenes and microRNAs which were enriched in immune signaling pathways, by RT-PCR (Table 1). Although the differential expression of microRNA mmu-miR-146a-5p, mmu-miR-181-5p have no corresponding immune pathways, they play an important role in the immune system[44, 45]. We also validated the unigenes enriched in the Toll-like receptor signaling pathway. RT-PCR were conducted with an MX300P Spectrofluorometric Thermal cycler (Stratagene, US) and QPCRMasterMix SybrGreen (DBI, US), initiated with a 2 min incubation at 95℃, followed by 40 cycles of 94℃ for 20 s; 58℃ for 20 s. The relative RNA expression levels were normalized by GAPDH.

**Table 1:** The immune pathways—Related microRNA of *Sarcoptes scabiei*.

Pathway name	KO identifier	microRNA
Toll-like receptor signaling pathway	ko04620	tca-miR-1175-5p
Fc gamma R-mediated phagocytosis	ko04666	hme-miR-190 cqu-miR-1175-5p aae-miR-1175-5p tca-miR-1175-5p isc-miR-276
Chemokine signaling pathway	ko04062	tca-miR-1175-5p
Leukocyte transendothelial migration	ko04670	isc-miR-276 tca-miR-71-5p
Apoptosis	ko04210	hme-miR-190

## Results

**Fig 1:**
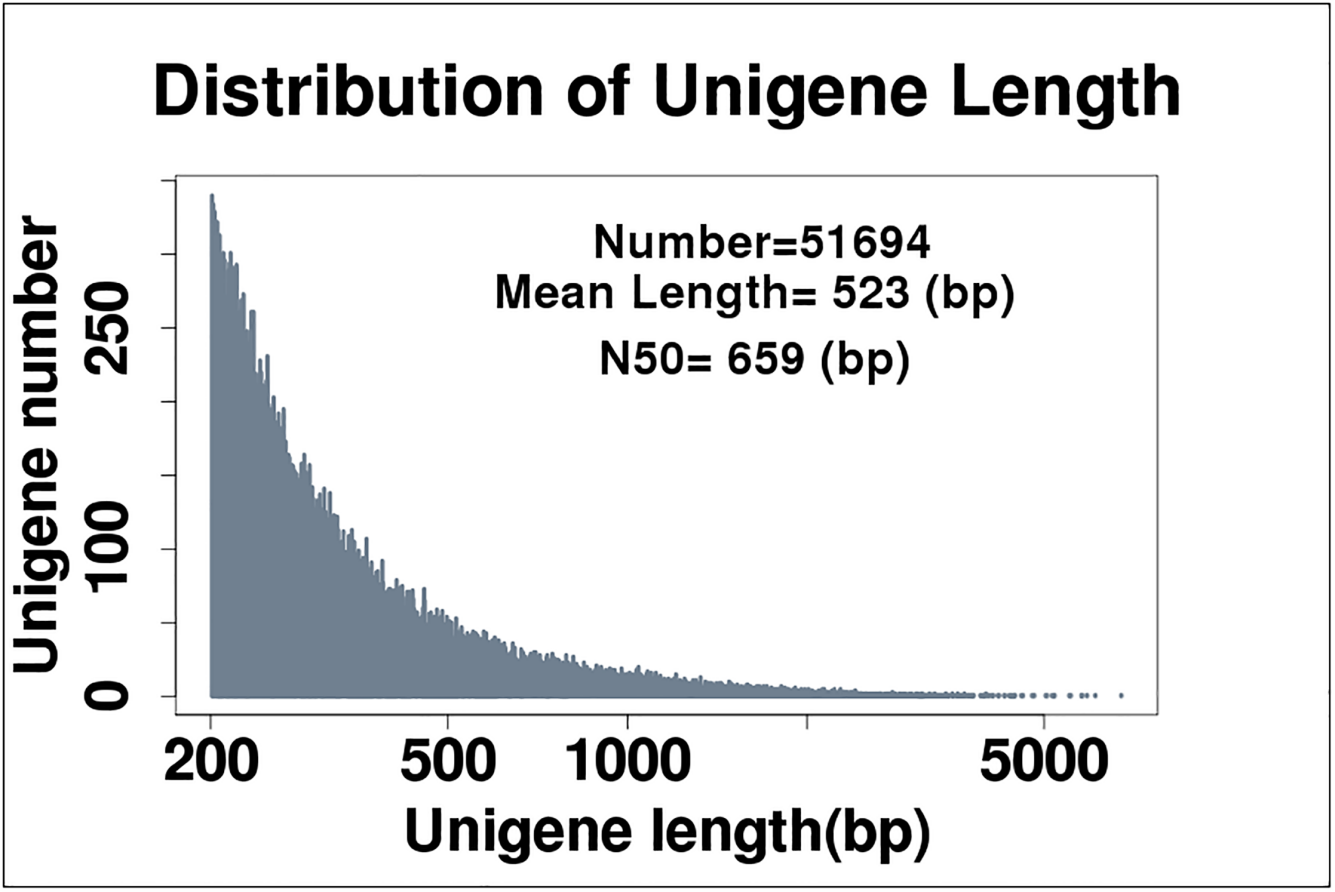
Distribution of the number of unigenes and read length in *S*.*scabiei* samples.

A total of 47,866,350 raw sequence reads were generated from the starved *S*. *scabiei* with 43,425,649 clean reads after filtering. *S*. *scabiei* embedded in host skin and infected host skin samples resulted in 69,307,202 raw reads, and 63,528,716 clean reads after filtering. Skin from uninfected rabbits resulted in 41,684,036 raw reads, and 38,114,803 clean reads after filtering. A total of 51,694 unigenes were detected after assembly (Fig 1) and compared, using cluster analysis, to 9,290 reference unigenes obtained from the Clusters of Orthologous Groups of proteins (COGs) database (http://www.ncbi.nlm.nih.gov/) (S1 Fig). Of these, 14,198 (27.5%) clustered with known gene functions (S1 Fig). The majority of reads, 62.99% and 64.87%, mapped to coding sequences for both infected and uninfected rabbits, respectively (S2A and S2B Fig).

Of the effective microRNA 81–84% mapped to the reference genome (S2 Table) with an average of 36% constituting uniquely mapped reads, and the majority mapping to intergenic regions (S2C and S2D Fig). The clean reads of microRNA were compared to Rfam database using bowtie program, and classified the result according to the type of RNA, the clean reads compared to 55–58%microRNA.

The transcriptome and microRNA datasets (raw reads) are available at the NCBI Bio Project with the ID: PRJNA320671 and Sequence Read Archive under the following accession numbers: SRS1432790, SRS1424964, SRS1432788.

### Differential expression analysis

We identified 4,276 differentially expressed unigenes in *S*. *scabiei*, with 3,215 up-regulated and 1061 down-regulated (S3A Fig). The gene function clustering results of *S*. *scabiei* showed that unigenes related to development, such as mitotic cycle and embryonic development, were up-regulated (S3 Table), and protein kinase activity and ATP combination, phosphorylation and redox enzyme activity were down-regulated.

Between infected and uninfected host rabbits there were a total of 467 differentially expressed genes, with 295 up-regulated and 172 down-regulated (S3B Fig). The gene function clustering results of infected and uninfected rabbit hosts demonstrated that cell adhesion, cell surface receptors, cytokine production, interaction between cells, immune system regulation, T cell proliferation differentiation, leukocyte proliferation differentiation and lymphocyte activatation, and ion related unigenes were up-regulated (S4 Table). Cytokine activation, immunoglobulin, signal peptide synthesis related genes were up-regulated and host energy metabolism related genes were down-regulated.

### MicroRNA

A total of 2,286,338 raw microRNA reads of starved *S*. *scabiei* resulted in 1,868,405 clean reads after filtering. Host embedded *S*. *scabiei* and infected host skin samples resulted in 6,419,608 raw reads, and 4,703,417 after filtering. Uninfected rabbit skin samples resulted in 6,307,144 raw reads, with 4,219,963 clean reads.

A comparison of uninfected host microRNAs to starved mite microRNAs revealed low similarity and 18 and 16 microRNAs were the same between host and parasite, respectively. There were 447 differentially expressed *S*. *scabiei* microRNAs, with 358 were up-regulated and 89 were down-regulated, and 18 differentially expressed host microRNAs, with five up-regulated and 13 down-regulated (S5 Table).

### RNA-seq and microRNA-seq comprehensive analysis

Using the predicted target unigenes of microRNA and differentially expressed microRNAs, we filtered target unigenes of *S*. *scabiei*. A total of 3,703 microRNA target genes were down-regulated, while 17,111 were up-regulated. Additionally, the potential target unigenes and mRNA-seq DEG overlap were analyzed and functional clustering results demonstrated that *S*. *scabiei* down-regulated microRNAs corresponding to up-regulated target unigenes were enriched in sugar and protein hydrolases, cell morphological differentiation, actin filament, and post-embryonic development signaling pathways (S6 Table). While hydrolytic enzymes play an important role in feeding and invasion of *S*. *scabiei*, indicating that the microRNAs are very important in regulating invasion and feeding of *S*. *scabiei*. Host down-regulated microRNAs corresponding to up-regulated target genes were enriched in synthesis of cell surface receptors and signal transduction, regulation of leukocyte, lymphocyte, T cell activation, and regulation of immune system signaling pathways (S7 Table).

### Validation of mRNA-seq and microRNA-seq results by RT-PCR

**Fig 2:**
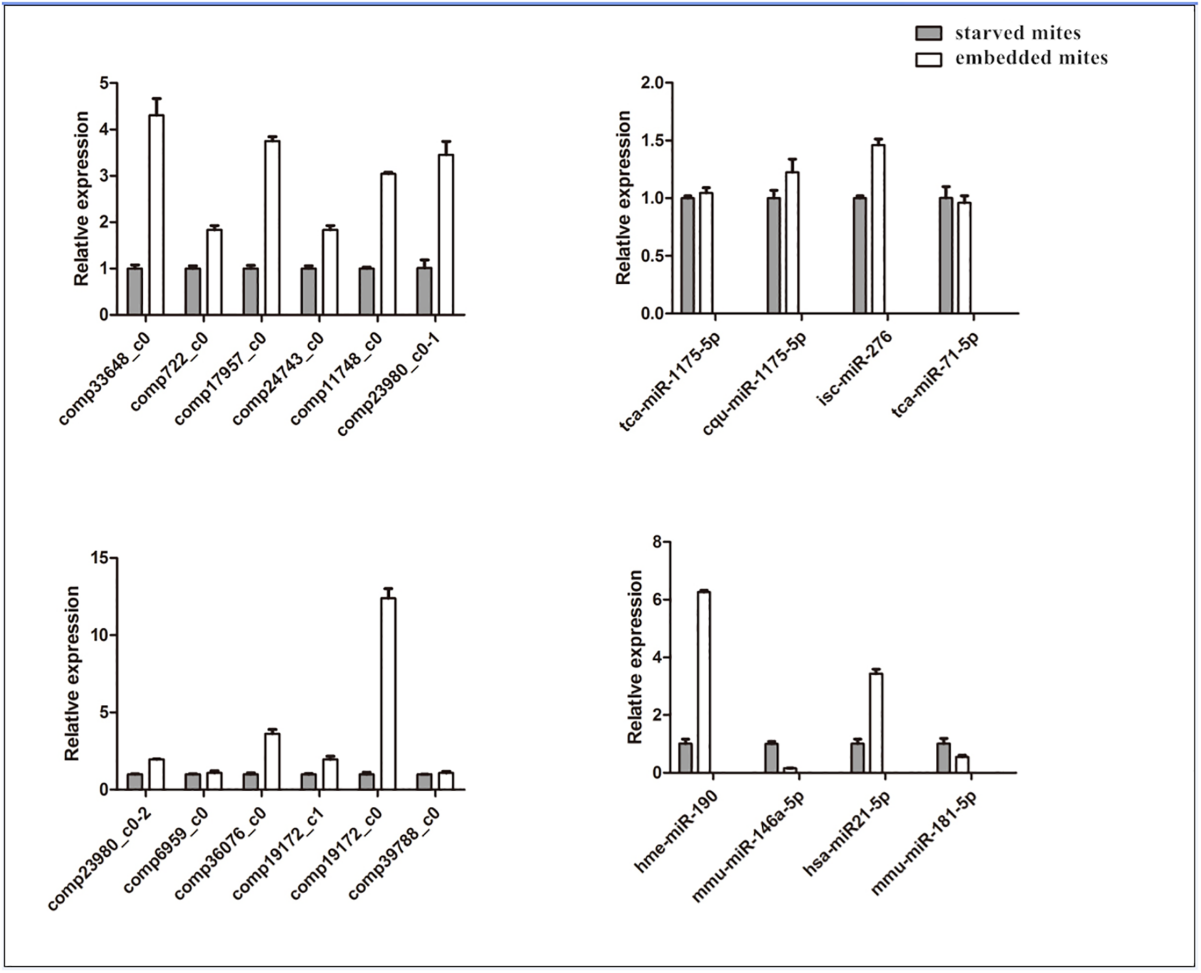
RT-PCR validation of the differentially expressed unigenes and microRNAs from Illumina sequencing. The relative expression levels of toll like receptor signaling pathway related unigenes and immune signaling pathways related microRNAs were determined by RT-PCR using cDNA as template.

The results of RT-PCR indicated differential expression of the analyzed microRNA and unigenes (Fig 2), consistent with the results of mRNA-seq and microRNA-seq data. This results validated expression data and verified the reliability and accuracy of our mRNA-seq and microRNA-seq analysis. Immune signaling pathway-related microRNAs and unigenes corresponding to Toll-like receptors signaling pathway were validated by RT-PCR (Table 1, S8 Table).

### Allergen and hydrolase genes

A total of 287 allergen genes from three species of mites were investigated for homologous sequences. We found 17 homologous sequences from *E*. *mayne* (S9A Table), 141 from *D*. *pteronyssinus* (S9B Table), and 580 from *D*. *farinae* (S9 Table). Additionally, we found 264 hydrolase unigenes of *S*. *scabie*i (S10 Table).

## Discussion

### Differential expression of genes

Scabies mites, *S*. *scabiei*, are obligate parasites that infect their host by burrowing into the skin. It is in the skin where they obtain their nutrients and undergo cell mitosis and embryonic development[15].

Mites burrow into skin and cause an intensely pruritic rash, crusted scabies may weaken the immunity of hosts, lead to skin damage on a large scale. *S*.*scabiei* can also facilitate secondary infection such as bacterial infections[46, 47]. Diagnosis depending on clinical symptoms is hard to distinguish scabies from hairless tinea, lice, crab lice and eczema because of the similar symptoms[48, 49]. Recent researches on immunity response among scabies mites and host have mainly focused on cytokines and antibodies[50–52], and high-throughput sequencing has not been applied to the analysis of the skin immune response. Since the affiliation between parasites and their hosts, and a significant a public health concern, this study explored the immune response of *S*. *scabiei* and host with transcriptome and microRNA comprehensive analysis.

After infection, host species exhibit loss of appetite, listlessness, inflammation, itching and crusting at the site of invasion[1, 7, 8]. We found that glycolysis, metabolic, and oxidation capacity-related genes of infected hosts were down-regulated, which may lead to some clinical symptoms. However, proliferation and differentiation of T cell and leukocyte, and activation of lymphocyte related unigenes were up-regulated, indicating that the host generates an immune response to resist invasion by the mite. The up-regulation of cytokine activation, immunoglobulin synthesis, and signal peptide synthesis related genes in infected hosts would lead to an increase secretion of cytokines and immunoglobulin in host serum.

We found that infection leading to up-regulation of *S*. *scabiei* unigenes related to increased cell proliferation and division, which would cause faster growth of the mite. Host immune response to infection are likely leading to down-regulation of the ATP combination, phosphorylation, redox enzyme activity and protein kinase activity related unigenes of *S*. *scabiei*. These unigenes are related to metabolism, membrane transport and intracellular signal transduction, suggesting that the metabolism and the body proliferation related genes of *S*. *scabiei* are inhibited by the immune response of the host.

Additionally, epidermal growth, vesicle mediated transport, and protein kinase mediated related unigenes were up-regulated, indicating that *S*. *scabiei* absorbs nutrients and excretes metabolic product related unigenes which are up-regulated due to *S*. *scabiei* feeding and grow within the host skin. *S*. *scabiei* secretions, feces, and the mites themselves, can be a trigger to the host immune response after invasion.

### Differential expression of microRNA

At the posttranscriptional level, microRNA plays a major role in the regulation of genome expression, regulating a variety of biological processes, such as maintenance of immune homeostasis and normal immune function[53]. MicroRNAs make a great importance in controlling the physiological, development and pathological processes, including cell proliferation, cellular differentiation and tumor generation[54–57]. MicroRNAs influence both innate and adaptive immune response[54] and may regulate the interaction of parasite and host, or drug resistance[58, 59]. MicroRNA impact the target gene in different ways, the immune pathway corresponding microRNA differential expression, immune pathway corresponding target genes differentially expression simultaneously according to the transcriptome and microRNA comprehensive analysis. Of the target unigenes of microRNAs enriched in immune signaling pathways we listed, three microRNAs were down-regulated. It showed that the expression of microRNA affects the expression of a target gene, which played a role in the activation or inhibition pathway. Indicating that microRNA play an important role in *S*.*scabiei* and host immune interactions in this study. The differential expression analysis of microRNAs provide a better understanding of the interaction between *S*. *scabiei* and host.

### Immune signaling pathway

In this study, *S*. *scabiei* and host DEGs were enriched in immune-related signaling pathways. *S*. *scabiei* DEGs were also enriched in JAK-STAT pathways, but neither in the immune deficiency pathway nor the toll signaling pathway, in spite of their role in the innate immune response[38, 60–62]. The JAK-STAT pathway influences, neutrophils and macrophages activation, inflammatory response and wound repair controlling has been indicated in the adaptive immune system by regulating the differentiation of B cells and T cells[63] and has been reported to be the main signaling pathway of the insect innate immune system. We found that eleven unigenes of toll-like receptors signaling pathway had been up-regulated, which indicated that this pathway is triggered upon infection and may be the main pathway in innate immune response. The expression of the eleven unigenes were validated by RT-PCR, the results showed that consistent unigenes expression has increased after infection, which has also indicated that the immune response was triggered because of the immune response of host. Our analysis of immune pathway in response to infection with *S*. *scabiei* has resulted in a better understanding of immune interaction of this parasite and host.

### Allergens of *Sarcoptes scabiei*

The biological, chemical and structural properties of dust mite allergens have been widely studied, about 21 dust mite allergens has a known allergen structure and can produce recombinant proteins, which may be useful to new diagnostic methods, or development of new drugs or vaccines[12, 64]. Dust mite and scabies mites have antigenic cross-reactivity[65, 66], therefore identifying homologous allergen genes in dust mites will allow us to identify these allergen genes in *S*. *scabiei*. A number of scabies mite allergen genes have known sequences homologies to house dust mite allergens including tropomyosin[14], serine proteases and cysteine proteases[21], glutathione S-transferase and apolipoprotein M-177[17]. 61 allergen unigenes had been predicted in transcriptome of *Sarcoptes scabiei canis*[67], and groups of allergen genes had been selected from the draft genome of the scabies mite[9]. Here we compared the mRNA-seq results with dust mite allergen and found 738 homologous allergen unigenes.

### Hydrolases of *Sarcoptes scabiei*

*S*. *scabiei* down-regulated microRNAs corresponding to up-regulated target unigenes which were enriched in sugar and protein hydrolase signaling pathways. This suggests that hydrolases are momentous in the immune interaction of *S*. *scabiei* and their hosts. Aspartic protease, produced by *S*. *scabiei*, plays a major role in the digestion of serum molecules and host skin[17]. Additionally, in *Psoroptes ovis* mites, a series of proteases are produced with anticoagulant effects that aid in degradation of fibrinogen, which ensures a continuous flow of host fluids during feeding[68]. Most of the house dust mite allergens are proteases, and the hydrolysis of these proteases promotes inflammation and aggravates allergic reactions[69–72]. Consequently hydrolases may play an important role in *S*. *scabiei* invasion and survival. Here we have compared the mRNA-seq results of *S*. *scabiei*, and built a hydrolase gene library which includes 264 hydrolase unigenes of *S*. *scabiei*.

By comparing the transcriptome and microRNA data we obtained a large number of differentially expressed genes and microRNA and the target genes of microRNA. The screened allergen genes will provide a support for further study on allergen functional characterization. Furthermore when comparing them to the hydrolase gene, some unigenes were both allergen and hydrolases, future studies identifying these allergens are warranted.

## Conclusion

With the comprehensive analysis of mRNA-seq and microRNA-seq results, we found that *S*.*scabiei* enhanced post-infected host immune function, decreased its metabolism. The immune response of host inhibited *S*.*scabiei* immune pathway, proliferation and metabolism signaling pathways. Simultaneously, we filtered a number of allergen and hydrolase unigenes, and it can be inferred that there existed unknown allergens in the hydrolase unigenes. Interestingly, it has been reported that the toll pathway, the immune deficiency pathway and JAK-STAT signaling pathway are the mainly signaling pathway of insect innate immune response. But in this study, the KEGG pathway analysis exhibited that the DEGs of *S*.*scabiei* enriched in JAK-STAT signaling pathway and Toll pathway but not in the immune deficiency pathway. Moreover the verification of the toll-like receptors signaling pathway indicated that it may be the mainly pathway in innate immune response of *S*.*scabiei*. The analysis of immune pathway, hydrolase and allergen will make a contribution to the research of *S*.*scabiei* pathogenesis, diagnosis and vaccines.

## Supporting information

S1 Fig Cluster analysis of RNA-seq detected unigenes compared to reference unigene sequences obtained from the COG database. A) Distribution of unigenes based on COG classifications, B) percent of sequences clustering based on biological processes, C) cellular components, and D) molecular function.
(RAR)

S2 Fig Distribution of host rabbit sequence reads aligning to the European rabbit reference genome. Sequence reads from A) infected host rabbits, and B) uninfected rabbits, C) infected host rabbits, and D) uninfected host rabbits.
(RAR)

S3 Fig Identification of differentially expressed unigenes. **A) Embedded mites and starved mites; B) infected host skin and uninfected host skin.** Comparison of DEG (DEG, red; not DEG, black) was performed in the R package, edgeR, with the.p-value set at p-value≤0.01; fold change≥2. The log concentration provides the overall concentration for a gene across the two groups being compared; the points on the left side signifies that genes were observed in only one group of replicate samples.
(RAR)

S1 Table Sample description. (DOCX)

S2 Table Mapping of clean reads on the rabbit’s genome. (DOCX)

S3 Table GO term analysis of differentially expressed unigenes of starved mites vs. embedded mites. (DOCX)

S4 Table GO term analysis of differentially expressed genes of infected host skin vs. uninfected host skin (DEG). (DOCX)

S5 Table Total number of differentially expressed microRNA (fold change≥2 or ≤0.01). (DOCX)

S6 Table GO term analysis of differentially expressed microRNA’ target unigenes of *Sarcoptes scabiei*. (DOCX)

S7 Table GO term analysis of differentially expressed microRNA’ target genes of host. (DOCX)

S8 Table Unigenes enrichment to the Toll-like receptors signaling pathway. (DOCX)

S9 Table Allergen homologous unigenes. **A) *Euroglyphus mayne* allergen homologous unigenes; B) *Dermatophagoides pteronyssinu* allergen homologous unigenes** (Only 20 of them are listed); **C) *Dermatophagoides farinae* allergen homologous unigenes** (Only 20 of them are listed).
(DOCX)

S10 Table Hydrolase homologous unigenes (Only 20 of them are listed). (DOCX)

## Abbreviations

*S.scabiei*: *Sarcoptes scabiei*
PRRs: pattern recognition receptors
KEGG: Kyoto Encyclopedia of Genes and Genomes
JAK: Janus Kinase
STAT: Signal transducers and activators of transcription
DEG: different expression unigenes
DEmicroRNA: different expression microRNA
RIG-I: retinoic acid-inducible gene I
TLR: toll like receptor
PAMP: pathogen-associated molecular patterns
