# Supplementary material for: Transcriptome-microRNA analysis of Sarcoptes scabiei and host immune response
Source: PLoS One. 2017 May 23;12(5):e0177733. doi: 10.1371/journal.pone.0177733 (PMC5441584; doi:10.1371/journal.pone.0177733)
Supplement: S1 Table — (DOCX) [file pone.0177733.s004.docx]

| **Sample ID** | **Sample name** | **Description** |
| --- | --- | --- |
| M | Free living mites | *Sarcoptes scabiei* only |
| MR | Embedded mites with infected host skin | *Sarcoptes scabiei* and Infected rabbit skin |
| R | Uninfected host skin | Healthy rabbit skin |

**S1 Table Sample description**
