# Supplementary material for: Transcriptome-microRNA analysis of Sarcoptes scabiei and host immune response
Source: PLoS One. 2017 May 23;12(5):e0177733. doi: 10.1371/journal.pone.0177733 (PMC5441584; doi:10.1371/journal.pone.0177733)
Supplement: S2 Table — (DOCX) [file pone.0177733.s005.docx]

**S2 Table Mapping of clean reads on the rabbit’s genome**

| **Sample** | **Input reads** | **Total mapped** | **Unique mapped** | **Multiple mapped** |
| --- | --- | --- | --- | --- |
| R | 4219963 | 3547822(84.07%) | 1560085(36.97%) | 1987737(47.10%) |
| MR | 4703417 | 3823495(81.03%) | 1683682(35.80%) | 2139813(45.49%) |
