# Supplementary material for: Transcriptome-microRNA analysis of Sarcoptes scabiei and host immune response
Source: PLoS One. 2017 May 23;12(5):e0177733. doi: 10.1371/journal.pone.0177733 (PMC5441584; doi:10.1371/journal.pone.0177733)
Supplement: S3 Table — (DOCX) [file pone.0177733.s006.docx]

**S3 Table GO term analysis of differentially expressed unigenes of starved mites vs. embedded mites**

| **Number** | **Up-cluster** | **Down-cluster** |
| --- | --- | --- |
| 1 | cytosolic ribosome, Ribosome, mitotic spindle elongation, ribonucleoprotein, cytosolic large ribosomal subunit, ribosomal protein, cytoskeleton organization, structural constituent of ribosome, microtubule cytoskeleton organization, mitotic cell cycle, non-membrane-bounded organelle, translation, M phase | Membrane, transmembrane, integral to membrane, intrinsic to membrane, transmembrane region |
| 2 | actin binding, actin filament-based process, actin cytoskeleton organization, actin filament organization | metal-binding, ion binding, cation binding, zinc, zinc-finger, transition metal ion binding, zinc ion binding |
| 3 | neuron differentiation, cellular component morphogenesis, cell projection organization, neuron development, neuron projection development and morphogenesis, neuron recognition, cell recognition, axon midline choice point recognition, dendrite development | nucleotide-binding, adenyl nucleotide binding, ATP binding, adenyl ribonucleotide binding, ribonucleotide binding |
| 4 | actin-binding, cytoskeletal protein binding, microtubule cytoskeleton | ank repeat, Ankyrin |
| 5 | regulation of organelle organization, regulation of actin cytoskeleton organization, regulation of actin filament-based process, regulation of cytoskeleton organization, regulation of actin filament length, regulation of actin polymerization or depolymerization | LIM domain, Zinc finger |
| 6 | epidermal cell differentiation and development, morphogenesis of a polarized epithelium, instar larval or pupal morphogenesis and development, post-embryonic morphogenesis and development hair cell differentiation, establishment of imaginal disc-derived wing hair orientation | Receptor, neurotransmitter binding, neurotransmitter  receptor activity |
| 7 | membrane invagination, endocytosis, phagocytosis, engulfment, membrane organization, vesicle-mediated transport | calcium ion binding, calcium, lipid binding |
| 8 | neuron differentiation, photoreceptor cell differentiation, eye photoreceptor cell differentiation, establishment or maintenance of cell polarity, eye morphogenesis, R7 cell differentiation, sensory organ development, Natural killer cell mediated cytotoxicity, Jak-STAT signaling pathway | Phosphotransferase, phosphorus metabolic process, phosphate metabolic process, kinase, phosphorylation, Proton acceptor, Protein kinase, ATP binding site, protein kinase activity, protein amino acid phosphorylation; |
| 9 | morphogenesis of an epithelium, epithelium development, dorsal closure, stress-activated protein kinase signaling pathway, embryonic development via the syncytial blastoderm, embryonic development ending in birth or egg hatching, embryonic morphogenesis | Oxidoreductase, iron ion binding, oxidation reduction; |
| 10 | cell adhesion, cell-cell adhesion, signal peptide | protein transport, organelle membrane |
