# Supplementary material for: Transcriptome-microRNA analysis of Sarcoptes scabiei and host immune response
Source: PLoS One. 2017 May 23;12(5):e0177733. doi: 10.1371/journal.pone.0177733 (PMC5441584; doi:10.1371/journal.pone.0177733)
Supplement: S4 Table — (DOCX) [file pone.0177733.s007.docx]

**S4 Table GO term analysis of differentially expressed genes of infected host skin vs. uninfected host skin (DEG).**

| **Number** | **Up cluster** | **Down cluster** |
| --- | --- | --- |
| 1 | disulfide bond, glycosylation sites signal peptide | shrinkage fibers, myofibril, sarcomere muscle protein, muscle contraction, muscle composition and structure, cytoskeletal actin cytoskeletal protein binding, myosin binding, myosin complex, Z film myosin Ⅱ complexes, disease mutation, the structure of the active molecule, dynein, no membrane organelles within the cell pack |
| 2 | signal peptides, secreted, extracellular domain | muscle organ development, striated muscle contraction, heart disease, muscle fiber composition, striated muscle tissue development, actin structure, actin head, and morphology related to cellular components, myocardial cell differentiation and development, cardiac contraction, blood circulation, acetyl of skeletal muscle organ development, hypertrophic cardiomyopathy |
| 3 | defense response, inflammatory response, hit reply | thick filaments, myosin movement, myosin heavy chain dynein, methylation, thick filaments of striated muscle, tight fitting, sliding filaments, myosin head, the calcium binding domain protein, viral myocarditis, nuclear nucleotide-binding, ATP binding, intracellular transport |
| 4 | cytokines, cytokine activity, cytokine receptor interaction; | striated muscle contraction regulation, regulation of muscle contraction, ATP activity |
| 5 | outside the plasma membrane domains, cell surface immunoglobulin, vacuoles, cell adhesion molecules | skeletal muscle contraction, skeletal muscle, multicellular organisms motion |
| 6 | extracellular, cytoplasmic, transmembrane region, the inner membrane, plasma membrane intact, intrinsic membrane | immunoglobulin type 2, IGc2, fibronectin type 3 sub-domain, fibronectin Ⅲ, immunoglobulin Ribbon |
| 7 | regulation of T cell activation and proliferation of white blood cell activity and proliferation of regulation, regulation of lymphocyte activation and proliferation, T cell activation and proliferation of negative regulation of lymphocyte activation and proliferation of negative control, a negative regulator of the immune system processes, negative regulation of cell proliferation monocytes, white blood cells activation negative regulation | cardiac contractility, EF-Hand, blood circulatory system, calcium binding, Calcium-binding EF-hand, signaling mechanisms, cell differentiation, chromosome segregation, focal adhesion, leukocyte migration |
| 8 | leukocyte activation, a positive regulator of cell activation, T-cell selection, activation and differentiation of T cells, the immune system is regulated lymphocyte activation, bacterial immune response, regulation of immune T cells, immune from the immunoglobulin family receptors, somatic reconstruction, hematopoiesis, signal conditioning calcium ion-mediated immune response process control, blood-forming organs or lymphoid organ development, immune system development, lipopolysaccharide response | gluconeogenesis, glucose metabolism, have been synthesized sugars, monosaccharides synthesis, metabolism of pyruvate, ethanol synthesis, monosaccharides metabolism, carbohydrate synthesis, glycolysis, insulin signaling, oxidative capacity, cell sap |
| 9 | positive regulation of cell activation, T cell activation and differentiation of positive regulation, leukocyte activation positive control, primary immunodeficiency, positive regulation of lymphocyte activation and differentiation, differentiation of T cells in the thymus positive regulation, cell differentiation and development positive regulation | Calmodulin-binding, Glycyl lysine isopeptide, iso-peptide bonds, undifferentiated B cell lymphoma effect |
| 10 | carbohydrate binding, combine sugar, C-type lectin, signal anchor, Ⅱ antifreeze protein, conserved regions | sarcoplasmic reticulum calcium automatic adjustment function, metal ion homeostasis calcium ion transport, chemical cell homeostasis cation homeostasis of calcium signaling pathway |
