# Supplementary material for: Transcriptome-microRNA analysis of Sarcoptes scabiei and host immune response
Source: PLoS One. 2017 May 23;12(5):e0177733. doi: 10.1371/journal.pone.0177733 (PMC5441584; doi:10.1371/journal.pone.0177733)
Supplement: S5 Table — (DOCX) [file pone.0177733.s008.docx]

**S5 Table Total number of differentially expressed microRNA (fold change≥2 or ≤0.01)**

| **DEmicroRNA sample** | **Total microRNA** | **P-value.** | **Down regulated microRNA NO.** | **Up regulated microRNA NO.** | **Total DEmicroRNA** |
| --- | --- | --- | --- | --- | --- |
| M Vs MR | 2285 | 0.01 | 358 | 89 | 447 |
| R Vs MR | 293 | 0.01 | 13 | 5 | 18 |
