# Supplementary material for: Transcriptome-microRNA analysis of Sarcoptes scabiei and host immune response
Source: PLoS One. 2017 May 23;12(5):e0177733. doi: 10.1371/journal.pone.0177733 (PMC5441584; doi:10.1371/journal.pone.0177733)
Supplement: S6 Table — (DOCX) [file pone.0177733.s009.docx]

**S6 Table GO term analysis of differentially expressed microRNA’ target unigenes of *Sarcoptes scabiei***

| **Number** | **Down-regulated microRNA’ target unigene (up-regulated DEG) cluster** | **Up-regulated microRNA’ target unigene (down-regulated DEG) cluster** |
| --- | --- | --- |
| 1 | neuronal differentiation cytomorphology，associated with neuronal cell morphological development，cell recognition，cell movement，alternative splicing，axon guidance，plasmalemma | activation of specific transcription factors，transcription regulatory factors activation，RNA metabolism activation，DNA combination |
| 2 | epidermal growth factor，calcium binding，conserved domain |  |
| 3 | tracheal system development, bacterium tube morphological development, cell adhesion, epithelial cell morphogenesis, morphological differentiation of adult wing, attachments form development, post-embryonic development |  |
| 4 | cytosol ribosomes, cell cycle, ribosomal subunit, mitotic spindle, ribosomal proteins, microtubules and cytoskeleton organization, cell membrane organelles |  |
| 5 | glycosidase, lysosome, hydrolytic enzymes |  |
| 6 | macromolecule metabolic regulation, transcription regulation is expression, nitrogen metabolism regulation, cell biosynthesis are regulation, RNA metabolism regulation |  |
| 7 | undifferentiated B cell lymphoma reproductive pathway, protein metabolism, cell macromolecules metabolic process, protein hydrolysis |  |
| 8 | cell adhesion, cell movement, phosphate metabolic process, plasma membrane, glycoprotein, alternative splicing |  |
| 9 | RNA metabolism regulation and activation of transcription factors, in combination with DNA |  |
| 10 | metal ion binding，cation binding |  |
