# Supplementary material for: Transcriptome-microRNA analysis of Sarcoptes scabiei and host immune response
Source: PLoS One. 2017 May 23;12(5):e0177733. doi: 10.1371/journal.pone.0177733 (PMC5441584; doi:10.1371/journal.pone.0177733)
Supplement: S7 Table — (DOCX) [file pone.0177733.s010.docx]

**S7 Table GO term analysis of differentially expressed microRNA’ target genes of host**

| **Number** | **Down-regulated microRNA’ target gene (up-regulated DEG) cluster** | **Up-regulated microRNA’ target gene (down-regulated DEG) cluster** |
| --- | --- | --- |
| 1 | cytoskeletal proteins, actin binding | glycoprotein, glycosylation sites (N- acetylglucosamine), a disulfide bond, a signal peptide |
| 2 | muscle protein, sarcomere contraction fibers without membrane organelles within the cell | immune response, defense response, innate immune response |
| 3 | glycosylation site, glycoprotein, disulfide bonds, the signal peptide, the cytoplasmic, transmembrane, native membrane | extracellular, cytoplasmic, transmembrane domain, membrane, plasma membrane, native cell membrane, transmembrane |
| 4 | bound metal ion, cation binding zinc ions bound | cell surface lymphocyte activity, receptor |
| 5 |  | secreted, extracellular domain |
| 6 |  | carbohydrate binding, immune response, lectins, sugar-binding, C-type lectins, conserved sites, the signal anchor |
| 7 |  | Immune response, Discovery biostimulation, bacterial detection, identification receptor activity, external stimuli Discovery, bacterial infection reaction, polysaccharide conjugate, glycosaminoglycan binding |
| 8 |  | the cell surface, an outer membrane, V-type immunoglobulin, T cell activity regulation of lymphocyte activation and differentiation of negative regulation, cell adhesion molecules (CAMs), a negative regulator of leukocyte activation, the immunoglobulin fold, negative regulation of the immune system, T cells activation and differentiation of negative regulation, T cell activation positive regulation, regulation of lymphocyte activation positive, positive regulation of leukocyte activation, T cell receptor signaling pathway |
| 9 |  | cytokine binding, cytokine receptor activation, cytokine and cytokine receptor interaction, growth factor binding |
| 10 |  | lymphocyte adhesion and oozing, exudation and neutrophil adhesion, cells and molecules involved in the local inflammatory response |
