# Supplementary material for: Transcriptome-microRNA analysis of Sarcoptes scabiei and host immune response
Source: PLoS One. 2017 May 23;12(5):e0177733. doi: 10.1371/journal.pone.0177733 (PMC5441584; doi:10.1371/journal.pone.0177733)
Supplement: S8 Table — (DOCX) [file pone.0177733.s011.docx]

**S8 Table Unigenes Enrichment to the Toll-like receptors signaling pathway**

| **KO identifier** | **Gene** | **Unigene** |
| --- | --- | --- |
| K02649，K00922 | P13K | comp722_c0 |
| K04398 | CASP8 | 19172_c0,1972_c1 |
| K04440 | JNK | 39788_c0 |
| K04734 | IκBα | 24743_c0,11748_c0 |
| K11220 | STAT1 | 33648_c0 |
| K04379，K04448 | AP-1 | 17957_c0 |
| K04519 | IL-1β | 36076_c0 |
| K10030 | IL-8 | 23980_c0-1,23980_c0-2 |
