# Supplementary material for: Transcriptome-microRNA analysis of Sarcoptes scabiei and host immune response
Source: PLoS One. 2017 May 23;12(5):e0177733. doi: 10.1371/journal.pone.0177733 (PMC5441584; doi:10.1371/journal.pone.0177733)
Supplement: S9 Table — A) Euroglyphus mayne allergen homologous unigenes; B) Dermatophagoides pteronyssinu allergen homologous unigenes (Only 20 of them are listed); C) Dermatophagoides farinae allergen homologous unigenes (Only 20 of them are listed). (DOCX) [file pone.0177733.s012.docx]

**S9A Table *Euroglyphus mayne* allergen homologous unigenes**

| **Unigenes** | **Subject** | **Description** |
| --- | --- | --- |
| comp31167_c0_seq1 | gi\|14424447\|sp\|P25780.2\|PEPT1_EURMA | RecName: Full=Peptidase 1;  AltName: Full=Allergen Eur m I;  AltName: Full=Mite group 1 allergen  Eur m 1; AltName: Allergen=Eur m 1;  Flags: Precursor |
| comp25459_c1_seq1 | gi\|14424447\|sp\|P25780.2\|PEPT1_EURMA |  |
| comp27545_c0_seq1 | gi\|14424447\|sp\|P25780.2\|PEPT1_EURMA |  |
| comp3310_c0_seq1 | gi\|14424447\|sp\|P25780.2\|PEPT1_EURMA |  |
| comp24210_c0_seq1 | gi\|14424447\|sp\|P25780.2\|PEPT1_EURMA |  |
| comp30024_c1_seq3 | gi\|14424447\|sp\|P25780.2\|PEPT1_EURMA |  |
| comp8468_c0_seq1 | gi\|14424447\|sp\|P25780.2\|PEPT1_EURMA |  |
| comp30024_c1_seq1 | gi\|14424447\|sp\|P25780.2\|PEPT1_EURMA |  |
| comp31660_c0_seq1 | gi\|14424447\|sp\|P25780.2\|PEPT1_EURMA |  |
| comp3666_c0_seq1 | gi\|14424447\|sp\|P25780.2\|PEPT1_EURMA |  |
| comp39153_c0_seq1 | gi\|14424447\|sp\|P25780.2\|PEPT1_EURMA |  |
| comp30177_c3_seq1 | gi\|4377538\|emb\|CAA42677.1\| | putative cysteine protease, partial [Euroglyphus maynei] |
| comp14662_c0_seq1 | gi\|4377538\|emb\|CAA42677.1\| |  |
| comp31091_c0_seq1 | gi\|4377538\|emb\|CAA42677.1\| |  |
| comp15227_c0_seq1 | gi\|5059164\|gb\|AAD38943.1\|AF144061_1 | alpha-amylase precursor [Euroglyphus maynei] |
| comp32824_c0_seq1 | gi\|4377538\|emb\|CAA42677.1\| | putative cysteine protease, partial [Euroglyphus maynei] |
| comp19184_c1_seq1 | gi\|14423649\|sp\|Q9TZZ2.2\|ALL2_EURMA | RecName: Full=Mite group 2 allergen Eur m 2; AltName: Allergen=Eur m 2; Flags: Precursor |

**S9B Table *Dermatophagoides* pteronyssinu allergen homologous unigenes** (Only 20 of them are listed）

| **Unigenes** | **Subject** | **Description** |
| --- | --- | --- |
| comp20965_c0_seq1 | gi\|37778944\|gb\|AAO73464.1\| | HDM allergen [Dermatophagoides pteronyssinus] |
| comp30568_c0_seq1 | gi\|20385544\|gb\|AAM21322.1\|AF373221_1 | group 14 allergen protein, partial [Dermatophagoides pteronyssinus] |
| comp38618_c0_seq1 | gi\|31745576\|gb\|AAP57077.1\| | trypsin-like serine protease [Dermatophagoides pteronyssinus] |
| comp3745_c0_seq1 | gi\|156124992\|gb\|ABU50814.1\| | Der p 3 allergen [Dermatophagoides pteronyssinus] |
| comp15813_c0_seq1 | gi\|60920912\|gb\|AAX37328.1\| | glutathione transferase delta-like Dp7018E11 [Dermatophagoides pteronyssinus] |
| comp27505_c1_seq2 | gi\|67975089\|gb\|AAY84565.1\| | group 15 allergen protein short isoform [Dermatophagoides pteronyssinus] |
| comp23447_c0_seq1 | gi\|208970286\|gb\|ACI32128.1\| | tropomyosin [Dermatophagoides pteronyssinus] |
| comp25339_c0_seq1 | gi\|1170095\|sp\|P46419.1\|GSTM1_DERPT | RecName: Full=Glutathione S-transferase; AltName: Full=GST class-mu; AltName: Full=Major allergen Der p 8; AltName: Full=P dp 15; AltName: Allergen=Der p 8 |
| comp26094_c0_seq1 | gi\|60920878\|gb\|AAX37326.1\| | glutathione transferase mu class Dp7019C10 [Dermatophagoides pteronyssinus] |
| comp8195_c0_seq1 | gi\|60920912\|gb\|AAX37328.1\| | glutathione transferase delta-like Dp7018E11 [Dermatophagoides pteronyssinus] |
| comp29331_c0_seq4 | gi\|188485735\|gb\|ACD50950.1\| | Der p 20 allergen [Dermatophagoides pteronyssinus] |
| comp22266_c0_seq1 | gi\|20385544\|gb\|AAM21322.1\|AF373221_1 | group 14 allergen protein, partial [Dermatophagoides pteronyssinus] |
| comp25210_c0_seq1 | gi\|156124992\|gb\|ABU50814.1\| | Der p 3 allergen [Dermatophagoides pteronyssinus] |
| comp19417_c0_seq1 | gi\|156124992\|gb\|ABU50814.1\| | Der p 3 allergen [Dermatophagoides pteronyssinus] |
| comp24455_c0_seq1 | gi\|721683625\|gb\|AIX10299.1\| | ribosomal protein S4, partial [Dermatophagoides pteronyssinus] |
| comp33849_c0_seq1 | gi\|1170095\|sp\|P46419.1\|GSTM1_DERPT | RecName: Full=Glutathione S-transferase; AltName: Full=GST class-mu; AltName: Full=Major allergen Der p 8; AltName: Full=P dp 15; AltName: Allergen=Der p 8 |
| comp28043_c0_seq1 | gi\|156124992\|gb\|ABU50814.1\| | Der p 3 allergen [Dermatophagoides pteronyssinus] |
| comp28043_c0_seq1 | gi\|156124992\|gb\|ABU50814.1\| | Der p 3 allergen [Dermatophagoides pteronyssinus] |
| comp10963_c0_seq1 | gi\|156124992\|gb\|ABU50814.1\| | Der p 3 allergen [Dermatophagoides pteronyssinus] |
| comp19651_c1_seq1 | gi\|157696052\|gb\|ABV66255.1\| | Der p 1 allergen, partial [Dermatophagoides pteronyssinus] |

**S9C Table *Dermatophagoides farinae* allergen homologous unigenes** (Only 20 of them are listed）

| **Unigenes** | **Subject** | **Description** |
| --- | --- | --- |
| comp29126_c0_seq4 | gi\|685432804\|gb\|AIO08856.1\| | Der f 26 allergen [Dermatophagoides farinae] |
| comp22674_c0_seq1 | gi\|442565878\|gb\|AGC56219.1\| | ferritin [Dermatophagoides farinae] |
| comp30803_c0_seq3 | gi\|685432828\|gb\|AIO08868.1\| | Ves m 1-like allergen [Dermatophagoides farinae] |
| comp36468_c0_seq1 | gi\|685432794\|gb\|AIO08851.1\| | Der f 27 allergen [Dermatophagoides farinae] |
| comp19292_c1_seq1 | gi\|37958173\|gb\|AAP35081.1\| | Der f Alt a 10 allergen [Dermatophagoides farinae] |
| comp29201_c1_seq1 | gi\|685432794\|gb\|AIO08851.1\| | Der f 27 allergen [Dermatophagoides farinae] |
| comp12932_c0_seq1 | gi\|387178006\|gb\|AFJ68066.1\| | Der f 1 variant, partial [Dermatophagoides farinae] |
| comp30414_c10_seq1 | gi\|685432804\|gb\|AIO08856.1\| | Der f 26 allergen [Dermatophagoides farinae] |
| comp27139_c0_seq3 | gi\|305387429\|gb\|ADM52184.1\| | Der f 1 allergen, partial [Dermatophagoides farinae] |
| comp12998_c0_seq1 | gi\|685432788\|gb\|AIO08848.1\| | Der f 28 allergen [Dermatophagoides farinae] |
| comp27183_c3_seq1 | gi\|371927457\|pdb\|3RVV\|A | Chain A, Crystal Structure Of Der F 1 Complexed With Fab 4c1 |
| comp7078_c1_seq1 | gi\|685432828\|gb\|AIO08868.1\| | Ves m 1-like allergen [Dermatophagoides farinae] |
| comp22512_c1_seq1 | gi\|442565874\|gb\|AGC56217.1\| | translation elongation factor 2, partial [Dermatophagoides farinae] |
| comp30992_c0_seq1 | gi\|37785882\|gb\|AAP57093.1\| | DFP2 [Dermatophagoides farinae] |
| comp22203_c0_seq1 | gi\|218203820\|gb\|ACK76293.1\| | Der f 3 allergen [Dermatophagoides farinae] |
| comp27245_c1_seq1 | gi\|37958173\|gb\|AAP35081.1\| | Der f Alt a 10 allergen [Dermatophagoides farinae] |
| comp29593_c1_seq1 | gi\|27550039\|gb\|AAM19082.1\| | 60 kDa allergen Der f 18p [Dermatophagoides farinae] |
| comp29294_c1_seq1 | gi\|37785882\|gb\|AAP57093.1\| | DFP2 [Dermatophagoides farinae] |
| comp27000_c2_seq1 | gi\|37958175\|gb\|AAP35082.1\| | Der f Gal d 2 allergen [Dermatophagoides farinae] |
| comp30771_c2_seq1 | gi\|1314736\|gb\|AAA99805.1\| | Der f 3 mite allergen, partial [Dermatophagoides farinae] |
