# Supplementary material for: Transcriptome-microRNA analysis of Sarcoptes scabiei and host immune response
Source: PLoS One. 2017 May 23;12(5):e0177733. doi: 10.1371/journal.pone.0177733 (PMC5441584; doi:10.1371/journal.pone.0177733)
Supplement: S10 Table — (DOCX) [file pone.0177733.s013.docx]

**S10 Table Hydrolase homologous unigenes**  (Only 20 of them are listed）

| **Unigene** | **Description** | **GO No.** |
| --- | --- | --- |
| comp10433_c0_seq1 | xaa-pro aminopeptidase | 1 |
| comp10451_c0_seq1 | signal peptidase complex subunit 3 | 4 |
| comp1155_c0_seq1 | aminopeptidase n | 14 |
| comp12246_c0_seq1 | carboxypeptidase a1-like | 1 |
| comp12246_c1_seq1 | carboxypeptidase b-like | 3 |
| comp15484_c0_seq1 | carboxypeptidase b-like | 1 |
| comp15484_c1_seq1 | carboxypeptidase b-like | 1 |
| comp15545_c0_seq1 | methionine aminopeptidase | 4 |
| comp16107_c0_seq1 | probable carboxypeptidase -like | 2 |
| comp16857_c0_seq1 | aspartyl dipeptidase | 2 |
| comp16857_c1_seq1 | dipeptidase e | 1 |
| comp17031_c0_seq1 | signal peptide peptidase-like 2a | 7 |
| comp17267_c0_seq1 | gamma-glutamyl transpeptidase 1 | 1 |
| comp18090_c0_seq1 | cytosol aminopeptidase | 8 |
| comp18187_c0_seq1 | glutamyl aminopeptidase | 3 |
| comp18596_c0_seq1 | n-acetylated-alpha-linked acidic dipeptidase-like protein | 4 |
| comp19532_c0_seq1 | caspase apoptosis-related cysteine peptidase | 5 |
| comp19532_c1_seq1 | caspase apoptosis-related cysteine peptidase | 5 |
| comp19651_c1_seq1 | dipeptidyl peptidase 1 | 6 |
